# Latent Toxoplasma gondii Infection and Psychiatric Disorders With Emphasis on Schizophrenia: A Hospital-Based Cross-Sectional Study in Tripoli, Libya

**DOI:** 10.7759/cureus.115853

**Published:** 2026-09-06

**Authors:** Mohamed Salem Lashal, Ahlam Masaud Ellafi, Sajeda Khalid Kareem, Montaha Fathi Bshiwa, Maysa Mohamed Elghadamsi, Salih Faysil Alezabi, Safa Almahdi Mustafa Sharfudeen, Hajir Ali Dakheel

**Affiliations:** 1 Department of Parasitology, Faculty of Medicine⁠, University of Tripoli, Tripoli, LBY; 2 Department of Histology and Medical Genetics, Faculty of Medicine, University of Tripoli, Tripoli, LBY; 3 College of Medicine, Faculty of Medicine, University of Tripoli, Tripoli, LBY; 4 Department of Community and Family Medicine, Faculty of Medicine⁠, University of Tripoli, Tripoli, LBY

**Keywords:** latent toxoplasmosis, libya, psychiatric disorders, schizophrenia, toxoplasma gondii

## Abstract

Background: Internationally, latent *Toxoplasma gondii* infection has been associated with the pathophysiology of psychiatric disorders, especially schizophrenia; however, data from Libya are extremely limited.

Objective: This study sought to examine the association between latent *T. gondii* infection and psychiatric disorders in patients at Al-Razi Hospital in Tripoli, Libya.

Methods: A cross-sectional study was performed involving 74 patients diagnosed with psychiatric disorders. Demographic and clinical data were gathered, encompassing a history of feline exposure and chronic illness. Serological testing for *T. gondii* IgG antibodies was performed, and chi-square tests and multinomial logistic regression analysis were used to evaluate the association between *T. gondii* seropositivity and psychiatric diagnostic categories.

Results: The average age of the participants was 39.46 ± 10.94 years, and 71 (95.9%) of them were men. Schizophrenia (58, 78.4%), other psychosis (11, 14.9%), bipolar disorder (4, 5.4%), and depression (1, 1.4%) were the most common mental illnesses. In total, 25 (33.8%) of the patients tested positive for *T. gondii* IgG antibodies. There were no significant links between seropositivity and age, gender, chronic illness, or contact with cats. Multinomial logistic regression demonstrated a significant association between *T. gondii* IgG seropositivity and schizophrenia (p < 0.001); however, this finding should be interpreted cautiously due to sparse data and instability of regression estimates.

Conclusion: Latent *T. gondii* infection is significantly associated with schizophrenia in psychiatric patients in Libya. These results emphasize the possible involvement of *T. gondii* in the pathophysiology of schizophrenia and stress the necessity for additional extensive, longitudinal studies to elucidate underlying mechanisms and investigate potential preventive or therapeutic strategies.

## Introduction

*Toxoplasma gondii* (*T. gondii*) is an obligate intracellular protozoan parasite and the etiological agent of toxoplasmosis, representing one of the most prevalent zoonotic parasitic infections worldwide [1]. It is estimated that approximately one-third of the global human population carries a latent infection [1]. The parasite exhibits a complex lifecycle, with felids serving as the definitive hosts and humans, along with other warm-blooded animals, acting as intermediate hosts [2]. Human transmission primarily occurs through ingestion of soil, water, or food contaminated with sporulated oocysts shed in feline feces, or through consumption of undercooked meat containing tissue cysts [2]. Although primary infection is typically asymptomatic in immunocompetent individuals, toxoplasmosis can precipitate severe clinical outcomes in immunocompromised patients and pregnant women due to congenital transmission and neurological complications [3]. The global seroprevalence of *T. gondii* varies substantially, heavily influenced by environmental, climatic, cultural, and socioeconomic determinants. Elevated seroprevalence rates are frequently documented in developing regions, primarily attributed to increased exposure to contaminated environmental sources. In the Middle East and North Africa (MENA) region, climatic conditions and specific lifestyle practices further facilitate parasite transmission. In Libya, previous studies have demonstrated notable *T. gondii *seroprevalence across diverse demographics, particularly among pregnant women and the general population [4-6]. Nevertheless, despite this baseline epidemiological evidence, data regarding the association between latent *T. gondii* infection and neuropsychiatric disorders within the Libyan context remain remarkably sparse [4-6].

Biologically, acute *T. gondii* infection triggers a robust Th1-mediated immune response characterized by the secretion of interleukin-12 (IL-12) and interferon-gamma (IFN-γ), which restricts tachyzoite replication through indoleamine 2,3-dioxygenase (IDO)-induced tryptophan depletion. However, the parasite evades complete immune clearance by establishing chronic, latent tissue cysts within neural and glial cells of the central nervous system. Within the brain, latent infection induces chronic low-grade neuroinflammation via pro-inflammatory cytokines, including IL-1β, IL-6, and TNF-α, ultimately disrupting neurotransmitter homeostasis [7]. Furthermore, *T. gondii *encodes aromatic amino acid hydroxylases (AAH1/AAH2) that directly manipulate dopamine synthesis, potentially driving dopaminergic dysregulation [8]. The parasite also downregulates astrocytic glutamate transporter-1 (GLT-1), causing elevated extracellular glutamate levels and subsequent excitotoxicity [9]. Concurrently, activation of the kynurenine pathway leads to an accumulation of kynurenic acid, an N-methyl-D-aspartate (NMDA) receptor antagonist contributing to receptor hypofunction and cognitive deficits [10]. Additionally, infection compromises GABAergic inhibition by altering the localization of glutamate decarboxylase 67 (GAD67) and promoting microglial pruning of inhibitory synapses [11]. Collectively, these pathophysiological cascades dopamine excess, glutamatergic overactivity, NMDA receptor dysfunction, and impaired GABAergic tone mirror key neurochemical abnormalities implicated in major psychiatric illnesses [12].

A growing body of epidemiological and clinical literature supports a link between latent *T. gondii* infection, immunoglobulin G (IgG) seropositivity, and neuropsychiatric disorders, particularly schizophrenia and bipolar disorder [13,14]. Large-scale population-based investigations, alongside case-control studies conducted in countries such as Egypt, China, Finland, and the United Kingdom, have consistently reported significantly higher *T. gondii* IgG seroprevalence and psychotic-like symptoms among psychiatric cohorts compared to healthy controls [15-18]. Comprehensive meta-analyses reinforce these observations, indicating that individuals with schizophrenia are approximately two to three times more likely to be *T. gondii *seropositive [13,14]. Although findings concerning bipolar disorder remain somewhat less consistent across different populations [19-21], latent infection is increasingly recognized as a non-negligible environmental risk factor influencing susceptibility to other psychosis and affective spectrum disorders [22,23]. Despite global evidence implicating latent toxoplasmosis in the pathogenesis of psychiatric conditions, localized data evaluating this relationship in North Africa and specifically within Libya remain extremely limited. Addressing this knowledge gap is essential for understanding the clinical and epidemiological impact of *T. gondii* among psychiatric populations in the region. Therefore, this study aimed to investigate the association between latent *T. gondii* infection (determined by anti-*T. gondii* IgG enzyme-linked immunosorbent assay (ELISA) seropositivity) and psychiatric disorders among patients admitted to Al-Razi Psychiatric Hospital in Tripoli, Libya.

## Materials and methods

A hospital-based cross-sectional study was conducted at Al-Razi Psychiatric Hospital, the largest tertiary psychiatric facility in western Libya, providing both inpatient and outpatient mental health services. The hospital serves a wide catchment population from the western and southern regions of the country, making it a suitable setting for epidemiological assessment. Data collection was carried out over a two-month period from August to September 2024.

The study population comprised adult patients diagnosed with psychiatric disorders and admitted to the inpatient department during the study period. A total of 78 patients were initially recruited using a convenience sampling approach. After data verification and exclusion of incomplete records, 74 participants were included in the final analysis.

The study included patients aged 18 years or older, with a confirmed clinical diagnosis of a psychiatric disorder made by a specialist psychiatrist according to the International Classification of Diseases, 10th Revision (ICD-10) criteria [24], who provided informed consent to participate in the study and had complete clinical or laboratory data. Patients with co-occurring major neurological conditions or an unconfirmed psychiatric diagnosis, inability to provide informed consent due to severe cognitive impairment or acute psychiatric instability, or incomplete clinical or laboratory data were excluded.

Data were collected using a structured questionnaire and medical record review. Information obtained included sociodemographic characteristics (age, sex), clinical diagnosis of psychiatric disorder, history of chronic medical conditions, and history of contact with cats as a potential risk factor for *T. gondii* exposure.

Venous blood samples were collected from all participants under aseptic conditions and transported to the National Centre for Disease Control for analysis. Serum samples were tested for anti-*T. gondii* IgG antibodies using the ELISA technique. ELISA was performed according to the manufacturer’s instructions. The assay involves coating microtiter plates with *T. gondii* antigen, followed by incubation with patient serum samples. After washing, enzyme-linked secondary antibodies were added, and a chromogenic substrate was used to produce a measurable colour reaction proportional to antibody concentration. Optical density was measured spectrophotometrically, and results were interpreted as positive or negative based on established cutoff values.

Ethical approval for the study was obtained from the Ethics Committee of the National Centre for Disease Control under Ref No. NBC: 002.H-25.42⁠. Written informed consent was obtained from all participants prior to enrolment. All collected data were anonymized and handled confidentially in accordance with ethical and international research reporting standards, including the Strengthening the Reporting of Observational Studies in Epidemiology (STROBE) guidelines for observational studies.

Data were analysed using IBM SPSS Statistics for Windows, Version 26 (Released 2018; IBM Corp., Armonk, New York, United States). Descriptive statistics were used to summarize participant characteristics. Continuous variables were presented as mean ± standard deviation (SD), while categorical variables were expressed as frequencies and percentages. Bivariate analyses were conducted to assess the association between *T. gondii* IgG seropositivity and independent variables. The chi-square test (or Fisher’s exact test where appropriate) was used for categorical variables, and the Mann-Whitney U test was applied for non-normally distributed continuous variables.

To further examine the relationship between *T. gondii* IgG serostatus and psychiatric diagnoses, multinomial logistic regression analysis was performed. Psychiatric diagnoses were analyzed as separate categories (schizophrenia, other psychotic disorders (ICD-10 codes: F22, F23, F25), bipolar disorder, and depression), with depression specified as the reference category. Due to the small number of participants in some diagnostic categories, particularly bipolar disorder and depression, the regression estimates were interpreted cautiously. The model estimated odds ratios (ORs) with 95% confidence intervals (CIs), adjusting for age, sex, history of chronic illness, and history of cat contact. Statistical significance was defined as p < 0.05 was considered statistically significant.

## Results

A total of 74 patients participated in the study. The participants' average age was 39.46 ± 10.94 years, and 71 (95.9%) were men, while three (4.1%) were women. Of the participants, 24 (32.4%) indicated a history of contact with cats, while 50 (67.6%) reported no history of cat contact; 20 (27.0%) reported a history of chronic illness, while 54 (73.0%) did not. The most common psychiatric diagnosis was schizophrenia in 58 (78.4%) patients, followed by other psychosis in 11 (14.9%), bipolar disorder in four (5.4%), and depression in one (1.4%). Serological testing showed that 25 (33.8%) individuals were positive for *T. gondii* IgG antibodies (Table 1).

**Table 1:** Association Between Toxoplasma IgG Seropositivity and Selected Variables Among Psychiatric Patients * Mann-Whitney U test at the 95% confidence interval; ** Chi-square test at the 95% confidence interval.

Variable		Frequency of Toxoplasma IgG Positivity	Percentage	Pearson χ^2^	df	sig
Age (mean ± SD)		-	-	-	-	0.660*
Gender	Male	25	35.2	1.595	1	0.320**
	Female	0	0			
Chronic illness	Yes	8	40.0	0.473	1	0.583**
	No	17	31.5			
History of contact with cat	Yes	7	29.2	0.338	1	0.610**
	No	18	36.0			
Psychological illness	Schizophrenia cases	18	31.0	6.316	3	0.049**
	Bipolar cases	0	0			
	Depression cases	1	100.0			
	Other psychosis cases	6	54.5			

Upon analysing variables linked to *T. gondii* IgG seropositivity, no noteworthy associations were found with age (p = 0.660), gender (71 (95.9%) male; 3 (4.1%) female; p = 0.320), chronic illness (20 (27.0%) with chronic illness; 54 (73.0%) without; p = 0.583), or cat contact history (24 (32.4%) with contact; 50 (67.6%) without contact; p = 0.610). Nonetheless, there was a statistically significant association (p = 0.049) between *T. gondii* IgG seropositivity and the type of psychiatric condition. *T. gondii* IgG seropositivity was observed in 18 (31.0%) patients with schizophrenia, six (54.5%) with other psychosis, 0 (0%) with bipolar disorder, and one (100.0%) with depression (Table 1).

The relationship between *T. gondii* IgG seropositivity and major psychiatric diseases was evaluated using a multinomial logistic regression, with depression acting as the reference group. A statistically significant association was found between *T. gondii* IgG and schizophrenia, as indicated by the analysis (B = 16.235, p < 0.001). Although the corresponding OR was extremely high (Exp(B) = 11,234,698.6), this value should be interpreted cautiously due to numerical inflation caused by sparse data. In contrast, the associations between *T. gondii* IgG and bipolar disorder (B = 31.654, p = 0.985) or other psychosis (B = 15.254, p not computed) were not statistically significant. The presence of very large coefficients and undefined CIs suggests possible quasi-complete separation or small cell counts in some diagnostic categories. Therefore, the interpretation of results is based primarily on the direction and significance of the log-odds coefficients rather than the magnitude of the ORs. Overall, *T. gondii* IgG seropositivity appears to be significantly associated with schizophrenia but not with bipolar disorder or other psychosis (Table 2).

**Table 2:** Multinomial Logistic Regression Analysis of the Association Between Toxoplasma gondii IgG Seropositivity and Psychiatric Disorders (Reference Category: Depression)

Name of Disease	Predictor	B (Log-Odds)	Std. Error	Wald	df	Sig.	Exp(B)
Schizophrenia	Intercept	-13.344	1.270	110.334	1	<0.001	-
	Toxoplasma IgG	16.235	0.669	589.326	1	<0.001	11,234,698.6
Bipolar disorder	Intercept	-46.486	3324.288	0.000	1	0.989	-
	Toxoplasma IgG	31.654	1662.144	0.000	1	0.985	5.59 × 10^13^
Other psychosis	Intercept	-13.462	1.080	155.334	1	<0.001	-
	Toxoplasma IgG	15.254	-	-	1	-	4,213,011.991

## Discussion

This study provides preliminary evidence of an association between latent infection with *T. gondii* and schizophrenia among patients attending Al-Razi Psychiatric Hospital. Overall, 25 (33.8%) participants were seropositive for *T. gondii* IgG antibodies. Seropositivity was observed in 18 (31.0%) patients with schizophrenia, six (54.5%) with other psychosis, 0 (0%) with bipolar disorder, and one (100.0%) with depression. However, the small number of participants in some diagnostic categories, particularly depression (one, 1.4%), should be considered when interpreting these proportions. Given the cross-sectional design and limited sample size, these findings should be interpreted with caution. Importantly, the cross-sectional nature of this study design precludes establishing a direct causal relationship, and these results reflect an observed association rather than causation.

Several biological mechanisms may plausibly explain this observed association. Experimental and clinical evidence suggests that *T. gondii* infection may influence neurotransmitter systems, particularly through potential alterations in dopamine metabolism and neurotransmitter pathways, which are strongly implicated in the pathogenesis of schizophrenia [8]. In addition, chronic latent infection may induce a state of low-grade neuroinflammation characterized by elevated pro-inflammatory cytokines, potentially contributing to neuronal dysfunction [7]. The formation of intracellular cysts within neural tissue may influence neural function either directly or through immune-mediated pathways [12]. While these biological mechanisms provide a plausible framework, local epidemiological and environmental factors specific to the study region (Tripoli, Libya) may also influence these observed pathways and warrant further regional investigation.

Although the regression estimates were unstable because of sparse data, the observed direction of association is consistent with previous studies reporting higher *T. gondii* seropositivity among individuals with schizophrenia. In the present study, 18 (31.0%) of the 58 patients with schizophrenia were seropositive for *T. gondii* IgG. Early work by Yolken et al. reported increased exposure to *T. gondii* among patients with schizophrenia compared to controls [19]. Similarly, a systematic review and meta-analysis by Oncu-Oner and Can found that individuals with schizophrenia were significantly more likely to be seropositive for *T. gondii* [13]. More recent large-scale and population-based studies have also supported this association, suggesting a potential link between latent toxoplasmosis and psychotic disorders [17].

Importantly, this study contributes novel data from Libya, where epidemiological evidence regarding the relationship between toxoplasmosis and psychiatric disorders remains limited. As such, these findings help address a regional gap in the literature and provide a basis for future research in North Africa and similar settings.

Nevertheless, the observed association underscores the potential importance of considering infectious exposures, including latent *T. gondii* infection, in psychiatric research, particularly in endemic regions where evaluating cross-sectional study designs effectively allows researchers to establish the baseline prevalence of latent *T. gondii* infection within specific clinical psychiatric populations. Exploring the role of infectious and immunological factors in mental disorders may provide valuable insights into disease mechanisms and open new avenues for prevention and management.

Future research should focus on large-scale, longitudinal studies within North African populations, particularly in Libya, to clarify the temporal relationship and potential causality between *T. gondii* infection and psychiatric disorders. Such region-specific investigations are necessary to account for local risk factors, genetic susceptibility, and environmental interactions unique to this cohort, ultimately informing targeted regional strategies to reduce the burden of psychiatric morbidity.

Several limitations should be acknowledged. First, the cross-sectional design precludes establishing temporal or causal relationships between *T. gondii* infection and schizophrenia. Second, the use of convenience sampling and recruitment from a single tertiary psychiatric hospital may limit the generalizability of the findings. Third, the relatively small sample size and unequal distribution of diagnostic categories may have affected the stability and precision of the regression estimates. In particular, some diagnostic categories contained very few participants, resulting in unstable estimates and requiring cautious interpretation of the regression findings. Additionally, potential confounding factors, such as socioeconomic status, dietary habits, and environmental exposures, were not fully assessed. Finally, detection of *T. gondii* IgG antibodies indicates previous exposure but cannot distinguish between recent and remote infection. Future studies with larger, more balanced, and representative samples are needed to confirm these associations.

## Conclusions

This study showed a significant association between latent *T. gondii* infection and schizophrenia in patients at Al-Razi Hospital in Tripoli, Libya. Overall, a seroprevalence of 33.8% (25/74) of *T. gondii* IgG antibodies was found among the studied group, most of whom were seropositive patients with schizophrenia. The study adds valuable data to the literature on infection-associated psychiatric illness and highlights the relevance of the regional prevalence of latent toxoplasmosis in North Africa. Moreover, it suggests the possibility of a link between latent *T. gondii* infection and biological mechanisms underlying schizophrenia through immune, inflammatory, or neurochemical pathways. Despite the cross-sectional design and limitations in subgroup analyses, the study underscores the need for further regional investigations into the potential role of latent *T. gondii* infection in psychiatric disorders.
